# Reconstitution of Peripheral T Cells by Tissue-Derived CCR4^+^ Central Memory Cells Following HIV-1 Antiretroviral Therapy

**DOI:** 10.20411/pai.v1i2.129

**Published:** 2016-10-16

**Authors:** Yolanda D Mahnke, Kipper Fletez-Brant, Irini Sereti, Mario Roederer

**Affiliations:** 1 ImmunoTechnology Section, Vaccine Research Center, National Institutes of Allergy and Infectious Diseases (NIAID), National Institutes of Health (NIH), Bethesda, Maryland; 2 Immunology Core Laboratory, Vaccine Research Center, NIAID, NIH, Bethesda, Maryland; current affiliation for K.F.-B. is McKusick-Nathans Institute of Genetic Medicine, Johns Hopkins School of Medicine, Baltimore, Maryland; and Department of Biostastistics, Johns Hopkins Bloomberg School of Public Health, Baltimore, Maryland; 3 Clinical and Molecular Retrovirology Section, Laboratory of Immunoregulation, NIAID, NIH, Bethesda, Maryland

**Keywords:** immune reconstitution, T helper subsets, cytokines, polarization

## Abstract

**Background:**

Highly active antiretroviral therapy induces clinical benefits to HIV-1 infected individuals, which can be striking in those with progressive disease. Improved survival and decreased incidence of opportunistic infections go hand in hand with a suppression of the plasma viral load, an increase in peripheral CD4^+^ T-cell counts, as well as a reduction in the activation status of both CD4^+^ and CD8^+^ T cells.

**Methods:**

We investigated T-cell dynamics during ART by polychromatic flow cytometry in total as well as in HIV-1-specific CD4^+^ and CD8^+^ T cells in patients with advanced disease. We also measured gene expression by single cell transcriptomics to assess functional state.

**Results:**

The cytokine pattern of HIV-specific CD8^+^ T cells was not altered after ART, though their magnitude decreased significantly as the plasma viral load was suppressed to undetectable levels. Importantly, while CD4^+^ T cell numbers increased substantially during the first year, the population did not normalize: the increases were largely due to expansion of mucosal-derived CCR4^+^ CD4^+^ T_CM_; transcriptomic analysis revealed that these are not classical Th_2_-type cells.

**Conclusion:**

The apparent long-term normalization of CD4^+^ T-cell numbers following ART does not comprise a normal balance of functionally distinct cells, but results in a dramatic Th_2_ shift of the reconstituting immune system.

**STANDFIRST**

Following administration of antiretroviral therapy in advanced AIDS, a preponderance of the increased CD4 T cells in blood are Th_2_-biased, tissue-derived T cells, resulting in a strong imbalance of functional subsets compared to healthy adults.

## INTRODUCTION

Highly active antiretroviral therapy (ART) for the treatment of HIV-1 effectively suppresses plasma viral load (PVL) in a vast majority of individuals, as well as gradually restoring CD4^+^ T-cell numbers and function. The reconstitution of the CD4^+^ T-cell compartment in peripheral blood is essentially biphasic [1–3]. An early, rapid increase during the first three weeks [3, 4] may be due to redistribution of memory cells to the peripheral blood from sites of inflammation in the tissues; subsequently, a slower phase, evident after about three months of treatment, is at least in part due to *de novo* production of naïve CD4^+^ T cells from the thymus [3, 5], as well as improved T-cell survival [6, 7]. The frequency of proliferating (Ki67^+^) cells decreases in both the CD4^+^ and CD8^+^ T-cell compartments, with a transient increase after 6 months of therapy, mainly in CD4^+^ central memory (T_CM_) cells [8]. More advanced patients are reported to have proportionately faster reconstitution rates [9], though the lower the CD4^+^ T-cell nadir, the longer it takes to normalize this population [10]. More advanced patients are reported to have proportionately faster reconstitution rates, though the lower the CD4^+^ T-cell nadir, the longer it takes to normalize this population.

Beyond these basic changes, less is known about the evolution of the T-cell compartment's composition during ART. The most profound change described within the CD4^+^ and CD8^+^ T-cell lineages is an overall reduction in activation, as evidenced by loss of cells expressing CD38 [1, 9, 11] and HLA-DR [1, 11, 12], and a decrease in the mean fluorescence intensity (MFI) of CD38 on CD8^+^ T-cells [11, 13, 14]. These changes represent a (partial) normalization of the T-cells' pheno-type, towards that seen in healthy adults.

The HIV-specific T-cell response also changes dramatically following ART. Independent of the epitope, HIV-specific CD8^+^ T-cell responses exhibit an early, rapid decline, continued with slower kinetics once plasma viral loads have been suppressed to undetectable levels [15]. This reduction in magnitude is not accompanied by a change in the quality of the CD8^+^ T-cell response [16]; however, like the bulk T-cell compartment, the expression of CD38 and HLA-DR on HIV-1 Gag-specific T cells decreases during treatment [11].

Despite these apparent normalizations, treated subjects still have immune defects. Therefore, we set out to determine T-cell dynamics during ART in total, as well as in HIV-1 Gag-specific CD4^+^ and CD8^+^ T cells. We found an overall rebalancing in the differentiation of T cells, favoring less differentiated cells; in addition, molecules related to activation and functional suppression gradually decreased during treatment, trending towards levels observed in healthy individuals. In sharp contrast to these expected findings, the proportion of Th_2_-like CD4^+^ T_CM_ increased for at least six months following ART initiation, in a direction away from frequencies typical for healthy adults; these cells have characteristics of mucosal-derived cells. Therefore, ART-induced immune reconstitution does not necessarily lead to a normalization of the immune system as a whole, and may, for at least a year, lead to a state that is Th_2_-biased in nature.

## MATERIALS AND METHODS

### 

#### Ethics statement.

HIV-1^+^ subjects were enrolled and provided written informed consent at the Clinical Center of the National Institute of Allergy and Infectious Diseases, NIH, under a protocol approved by the NIAID Institutional Review Board. These studies were registered at www.clinicaltrials.gov as #NCT00557570 and #NCT00286767. Samples were coded; all analyses were performed blinded to identity.

#### Human subjects and sample collection.

The patient cohort has been described elsewhere [17]. Briefly, all patients (1) were ART-naïve (n = 56) or had interrupted treatment for at least one year (n = 4, plus n = 2 who had previously received brief mono- or dual therapy) with a viral rebound of > 10,000 copies/ml; (2) had ≤ 200 CD4^+^ T cells/μl at baseline; (3) suppressed their HIV-1 viral load to <500 copies/ml within one year of ART; and (4) had available peripheral blood mono-nuclear cell (PBMC) samples taken pre-ART as well as after 1, 3, 6, and 12 months of ART. Seventeen patients developed episodes of immune reconstitution inflammatory syndrome (IRIS; defined according to the AIDS Clinical Trials Group criteria, <https://actgnetwork.org/IRIS_Case_Definitions>) following commencement of ART, while 39 underwent uneventful immune reconstitution. PBMC from 12 healthy donors served as controls (Table 1).

**TABLE 1.** PATIENT COHORT CHARACTERISTICS

	HIV^+^	HIV^−^
*n*	56	12
age ^A,B^	37.2 (31.2-43.2)	36.4 (32.5-39.3)
male (%)	76.8	66.7
ethnicity (%)		
African	51.8	58.3
Asian	0.0	8.3
Caucasian	14.3	16.7
Hispanic or Latino	25.0	0.0
Native American or Alaska Native	1.8	0.0
mixed	7.1	16.7
ART component (%)		
NNRTI ^C^	64.3	
PI ^C^	35.7	
Time relative to ART initiation (months)		
pre-ART	-0.2 (-0.5-0)	
mo1	1 (0.9-1.2)	
mo3	3 (2.8-3.2)	
mo6	5.6 (5.6-6.1)	
mo12	12 (11.2-12.5)	

(A) HIV^+^: at ART initiation; HIV^−^: at time of PBMC sampling

(B) Median (IQR)

(C) NNRTI: non-nucleoside reverse-transcriptase inhibitors; PI: protease inhibitors

For the elucidation of T-helper subsets (Figures 5–6), PBMC of an additional 13 HIV-1^+^ individuals were sampled before, as well as one month, and 12 months after initiation of ART. Their clinical parameters were comparable to that of the main cohort, with the following medians and inter-quartile ranges pre-ART: 56 (20-77) CD4^+^ T cells/μl, 572 (469-744) CD8^+^ T cells/μl, 4.8 (4.5-5.4) log_10_ PVL; after 1 month of ART: 129 (101-152) CD4^+^ T cells/μl, 918 (589-1105) CD8^+^ T cells/μl, 2.3 (1.9-2.7) log_10_ PVL; and after 12 month of ART: 210 (199.8-264.5) CD4^+^ T cells/μl, 795.5 (555.5-950) CD8^+^ T cells/μl, 1.7 (1.7-1.7) log_10_ PVL. None of these patients experienced IRIS. PBMC from an additional 16 healthy donors served as controls for this part of the study.

#### Determination of plasma viral load, CD4^+^, and CD8^+^ cell counts.

Plasma HIV-1 viral loads (PVL), as well as CD4 and CD8 counts were determined in a laboratory operating under the Clinical Laboratory Improvement Amendment (CLIA). The plasma viral load was measured using the ultrasensitive Quantiplex HIV-1 bDNA version 3.0 (Bayer). CD4^+^ and CD8^+^ T-cell counts were determined by four-color flow cytometry. The BD Multitest (BD Biosciences) that was used includes the following Abs: CD3^FITC^ (clone SK7); CD4^APC^ (clone SK3); CD8^PE^ (clone SK1); and CD45^PerCP^ (clone 2D1). Samples were acquired on either FACSCalibur or FACSCanto (both BD Biosciences). CD4^+^ cell counts were calculated as percent of CD4^+^ CD3^+^ cells within CD45^+^ lymphocytes divided by 1% of the white blood cell count. The corresponding calculation was performed for CD8^+^ cell counts.

#### Sample preparation and Ag-stimulation.

Cryopreserved PBMC were thawed in pre-warmed RPMI 1640, 10% FCS, 2mM L-glutamine, 100 U/ml penicillin, and 100 μg/ml streptomycin (all from Gibco; this medium will hereafter be referred to as complete RPMI), in the presence of 20 μg/ml benzonase nuclease (Novagen). Cells were rested in complete RPMI for 4-6 hours at 37°C, 5% CO_2_ and either left unstimulated (mock control) or stimulated overnight in 200 μl complete RPMI with 2.5 μg/ml HIV-1 Gag peptide pool (NIH AIDS Research and Reference Reagent Program, Germantown MD) in the presence of anti-CD49d and anti-CD28^PE -Cy5^ mAb (BD Biosciences). Monensin and Brefeldin A (BD Biosciences) were added after 2 hours of stimulation. Healthy donor PBMC were stimulated with SEB (Sigma) to serve as a positive control.

#### Flow cytometry.

The reagent panels used in the present study are listed in Supplementary Table 1. All except the “Th subset” and “sorting” panels have been described in previous publications [17, 18]. The “Th subset” panel included the following additional reagents: CCR6^Ax488^ (clone TG7/ CCR6); CCR10^PE^ (clone 6588-5, both from BioLegend); CXCR3^PE-Cy5^ (clone 1C6/CXCR3); and HLA-DR^PE-Cy5.5^ (clone TÜ36, both from BD Biosciences). The “sorting” panel included the following additional reagents: CCR6^BV605^ (clone G034E3); CCR4^PE-Cy7^ (clone TG6/CCR4, both from BioLegend); CD4^APC^ (clone RPA-T4, BD Biosciences); as well as TCR-Vβ12 (clone VER2.32.1); TCR-Vβ14 (clone CAS1.1.1.3); and TCR-Vβ17 (clone E17.5F3.15.13) conjugated to FITC (Life Technologies) at the VRC; and TCR-Vβ1 (clone BL37.2); TCR-Vβ2 (clone MPD2D5); TCRVβ7 (clone ZOE); TCR-Vβ13.6 (clone JU74.3); TCR-Vβ16 (clone TAMAYA1.2); and TCR-Vβ22 (clone IMMU546) conjugated to Ax594 (Life Technologies) at the VRC. All unconjugated TCRVβ Abs were obtained from Beckman Coulter. For intracellular staining, cells were treated with BD Cytofix/Cytoperm Permeabilization Solution (BD Biosciences), except for the T_reg_ panel, where the Foxp3 Staining Buffer Set was employed (eBioscience). Data were acquired on an LSR II (BD Biosciences) using a high-throughput system (HTS).

#### Multi-parametric quantitative RT-PCR.

We largely followed the protocols set forth by Dominguez *et al*. [19]. Briefly, TaqMan^TM^ primer/probe sets (Life Technologies) were chosen for genes relevant for T-cell immunity, including those associated with cytokines, cytokine receptors, migration, proliferation, chemokines, cytolysis, transcription factors, activation, and costimulation (see Supplementary Table 2). Depending on subset abundance, 10-100 cells were sorted by fluorescence-activated cell sorting for assessment of gene expression in different CD4^+^ T-cell subsets (Supplementary Figure 5). Cells were sorted directly into cell culture plates containing 10μl of reaction mix (Invitrogen Cell Direct Kit^TM^, Life Technologies); the manufacturer's instructions were followed for reverse transcription (15min at 50°C) and cDNA synthesis (2min at 95°C; 15sec at 95°C; 4min at 60°C). Seventeen pre-amplification cycles were performed (15sec at 95°C; 4min 60°C).

Pre-amplified cDNA, and TaqMan^TM^ primer/probes were loaded onto a microfluidic chip (Fluidigm), and multi-parametric quantitative RT-PCR was performed using a Biomark^TM^ cycler (Fluidigm) as previously described [19].

#### Data analysis.

Flow cytometry data were analyzed using FlowJo (FlowJo, LLC), Pestle (NIAID, NIH; by M. Roederer), and SPICE 5.1 [20]. The gating scheme is identical to that used in our previous publications [17, 18]. All cytokine measurements were background subtracted, taking into account the frequency of cells producing cytokines in the absence of antigenic stimulation (mock control). For the phenotypic analysis of Ag-specific cells, only those samples with >10 cytokine-positive events and response magnitudes > 3x that of the corresponding mock control were considered. The mean fluorescence intensity (MFI) of CD38^+^ cells was calibrated using the experiment-matched internal control sample.

RT-PCR data were analyzed using JMP 11 (SAS), R 3.1, and Bioconductor [21]. Because varying cell numbers were sorted for RT-PCR of individual T-cell subsets, all samples were normalized to 50 cells. Relative gene expression levels or “expression threshold” (Et) are proportional to log_2_ RNA abundance and were calculated using the “cycle threshold” (Ct) obtained, where Et = 28-Ct [19]. The following genes, expressed by less than 10% of samples analyzed, were excluded from the analysis, as this could due to inefficient amplification: CXCL11; CXCR1; CXCR2; GPR44; IL5; IL9; and TGFB2.

#### “Th-ness” and Differentiation Index (DI).

Th-ness was defined as the posterior probability [22, 23] of class membership given by a support vector machine (SVM) [24, 25] trained to differentiate between all sorted healthy donor Th_1_- and Th_2_-like cells with radial basis kernel. All sorted CCR4^+^ T_CM_, CCR4^+^ non-T_CM_, CCR4^−^ T_CM_, and CCR4^−^ non-T_CM_ samples were then assigned a Th-ness value according to their gene expression pattern, indicating whether their phenotype was more similar to Th_1_- or Th_2_-like cells. Accuracy of the SVM was 90% for three-fold cross validation.

Each T-cell subset was assigned a weighting value as follows: T_NV_ = 0; T_CM*_ = 1; T_CM_ = 2; T_TM*_ = 3; T_TM_ = 4; T_EM_ = 5; T_TE*_ = 6; T_TE_ = 7. The DI is the average of the subset frequencies weighted by their respective values. As Nv cells do not contribute to a population's overall differentiation, they are assigned a weighting of 0. The weighted sum is then normalized by the maximum differentiation value (7) to derive a metric ranging from 0 to 1: DI = ((%T_NV_ * 0) + (%T_CM*_ * 1) + (%T_CM_ * 2) + (%T_TM*_ * 3) + (%T_TM_ * 4) + (%T_EM_ * 5) + (%T_TE*_ * 6) + (%T_TE_ * 7)) / 7 / 100%.

#### Statistical analysis.

Nonparametric tests were used for all analyses (SAS version 9.2); matched comparisons were performed where applicable. Changes from baseline (paired differences) were evaluated using the Sign test. Statistical comparisons of pie charts were performed in SPICE 5.1 software using 10,000 permutations [20]. Given the exploratory nature of this study, there was no adjustment for multiple comparisons; in most analyses, only *P*-values less than 0.01 are reported.

Differential expression analysis of genes assayed by RT-PCR was performed using Limma [26–28]. Results for CD103 were obtained via robust regression [29, 30]. All *P*-values from differential expression analyses were then pooled for control of false discovery rate [31]. Significance was then defined as an adjusted *P*-value less than 0.01.

## RESULTS

### Overall ART-Responsiveness

**Supplementary Figure 1.**
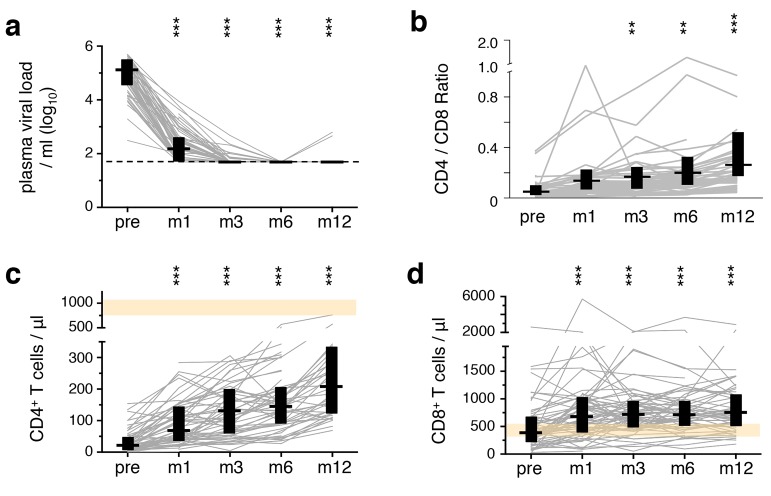
**Suppression of viral loads and recovery of T-cell counts on ART.** (A) The plasma viral load was determined by measuring HIV-1 RNA at each sampling time-point. The detection threshold was 50 copies/ml (indicated by the broken line), with rare exceptions of 100 or 500 copies/ ml. The CD4/CD8 ratio (B), and number of CD4^+^ T-cells (C) or CD8^+^ T-cells/μl (D) in peripheral blood are shown. Bars illustrate median values, while boxes show the inter-quartile range. Healthy ranges are indicated in orange (generated from 288 healthy donor PBMC processed in the testing laboratory). All time-points were compared to corresponding pre-ART measurements: * *P* < 0.01, ** *P* < 0.001, *** *P* < 0.0001.

Fifty-six HIV-1^+^ patients commenced ART when their CD4^+^ T-cell count was ≤ 200/μl. Phenotype and HIV-1 Gag reactivity of their PBMC-derived T cells were characterized before ART, as well as at 1, 3, 6, and 12 months after ART-initiation (Table 1). All patients rapidly responded to ART, evidenced by a 3-log suppression of the PVL within a month and to undetectable levels within 3 months (Supplementary Figure 1A). The CD4^+^ T-cell counts gradually increased during the time of follow-up. Though the increase was significant within 1 month of treatment, T-cell counts still remained largely below those observed in healthy adults at 1 year (Supplementary Figure 1B). CD8^+^ T-cell counts, which started in the range of levels typically observed in healthy adults, increased only during the first month of treatment (Supplementary Figure 1C) [18].

### Longitudinal Analysis of HIV-Specific T-Cell Responses During ART

**Figure 1.**
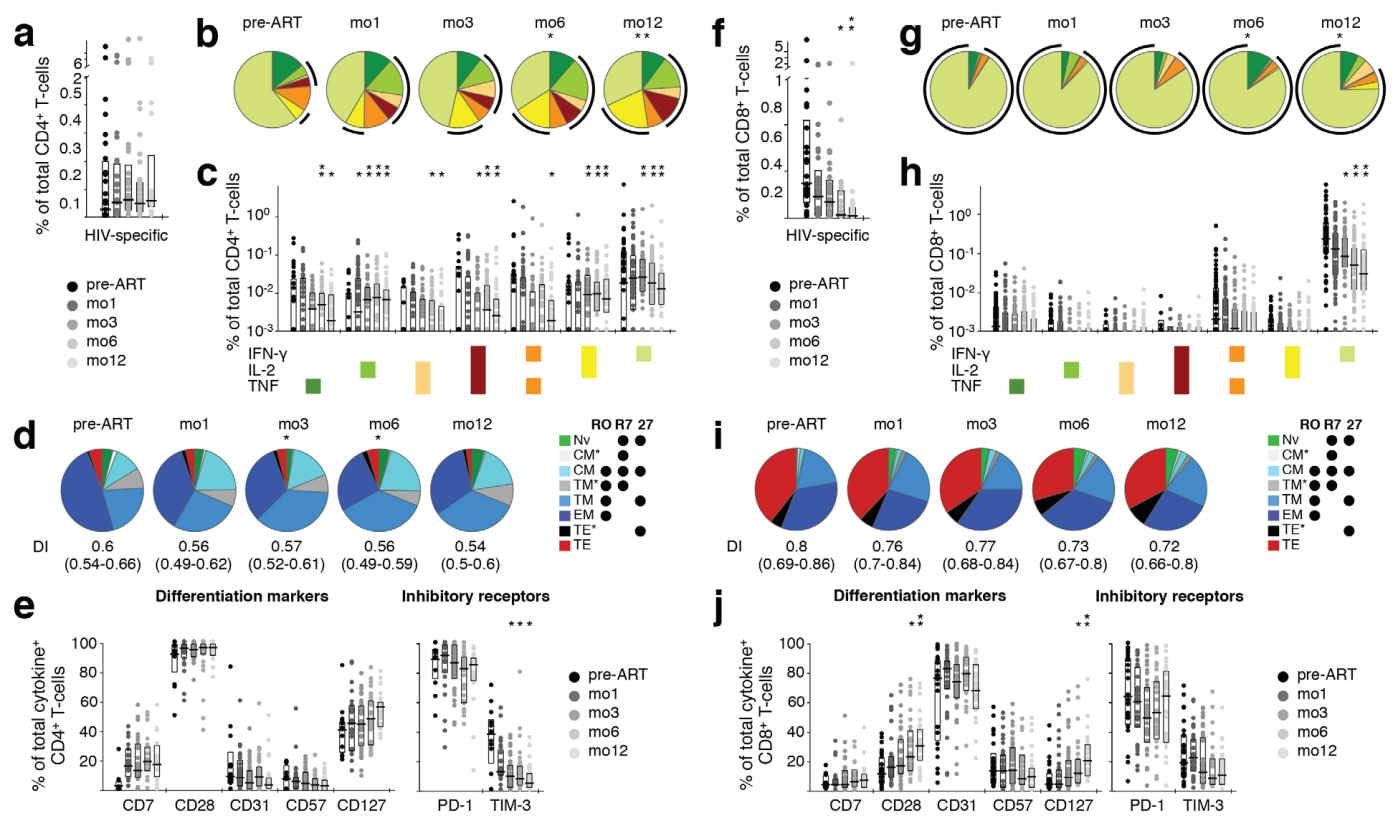
Longitudinal analysis of HIV-1 Gag-specific cytokine production by T cells and the phenotype of cytokine-producing cells. The effect of ART on HIV-1 Gag-reactive CD4^+^ (A-E) and CD8^+^ T cells (F-J) was determined in longitudinal PBMC samples of HIV-1^+^ patients. (A, F) Total response magnitude, measured by production of IFN-γ, IL-2, or TNF. (B, G) Cytokine pattern. Relative proportion of total HIV-1 Gag-reactive cells producing each possible combination of the cytokines measured. Black arcs indicate all IL-2 (B) or IFN-γ (G) producing cells. (C, H) Actual frequency of cells producing only IFN-γ, IL-2, or TNF, or any combination thereof. Potential phenotypic alterations occurring due to ART were explored in HIV-1 Gag-reactive CD4^+^ (D, E) and CD8^+^ T cells (I, J) in longitudinal PBMC samples of HIV-1^+^ patients. (D, I) Differentiation state. T-cell differentiation subsets of cytokine-positive cells were defined by expression of CD45RO (“RO”), CCR7 (“R7”), and CD27 (“27”). Differentiation indices (DI; medians and interquartile ranges) are indicated below each pie. (E, J) Phenotype. The frequency of cytokine-positive cells expressing differentiation markers (CD7, CD28, CD31, CD57, CD127) or inhibitory receptors (PD-1, TIM-3) was determined. Graphs show interquartile ranges, median bars, as well as individual data points. All time-points were compared to corresponding pre-ART measurements: *P <0 .01, **P < 0.001, ***P < 0.0001.

Even though the magnitude of the HIV-specific CD4^+^ T-cell response did not change within the first year of ART (Figure 1A), these cells became more polyfunctional (*i.e.*, producing two or three cytokines) over time, achieving statistical significance at late sampling time-points (6-12 months of ART, Figure 1B). This change in cytokine pattern was mainly due to increased IL-2 production (Figures 1B, C). The subset distribution within HIV-responsive CD4^+^ T cells was also affected by ART: as early as 3 months after commencing ART, less differentiated cells (T_CM_, T_TM_) increased, with a concomitant reduction in T_EM_ cells (Figure 1D). However, no significant change in the differentiation index (DI; see supplementary methods section) of HIV-1 Gag-specific CD4^+^ T cells was observed. Furthermore, the ART-induced reversal of other differentiation and inhibitory receptors' expression was much less dramatic for HIV-specific cells (Figure 1E) than that for total CD4^+^ T cells (see below). Though there were trends mirroring total CD4^+^ T cells, no statistically significant changes were observed in the phenotype of HIV-specific CD4^+^ T cells. Taken together, these data indicate that while the overall magnitude of HIV-specific CD4^+^ T cells remained unaffected by ART, these cells became mildly more enriched for less differentiated cells without changes in activation state.

**Supplementary Figure 2.**
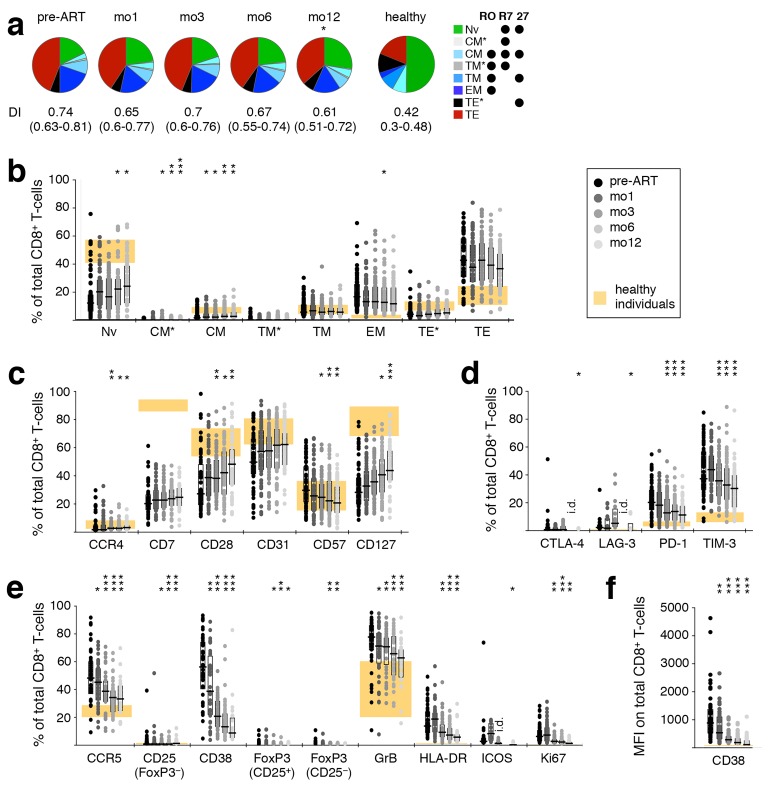
**ART-induced change towards less differentiated CD8^+^ T cells.** PBMC were sampled before, as well as after 1, 3, 6, and 12 months of ART. The distribution of differentiation stages (A) and frequency of individual differentiation subsets (B) was investigated in CD8^+^ T cells. T-cell differentiation subsets were defined by expression of CD45RO (“RO”), CCR7 (“R7”) and CD27 (“27”). Differentiation indices (DI; medians and interquartile ranges) are indicated next to each pie. The average T-cell differentiation profile as well as the interquartile range of the differentiation indices in healthy donors are shown. (C) Markers of Tcell differentiation. (D) Inhibitory receptors. (E) Markers of activation; GrB– Granzyme B. (F) Mean fluorescence intensity of CD38. Graphs show interquartile ranges, median bars, as well as individual data points. Orange areas represent the interquartile ranges of corresponding measurements in healthy individuals. All timepoints were compared to corresponding pre-ART measurements: * *P* < 0.01, ** *P* < 0.001, *** *P* < 0.0001.

In contrast, HIV-specific CD8^+^ T cells reacted very differently to ART than their CD4^+^ counterparts. As previously published [11, 16], there was a significant decrease in the magnitude of the CD8^+^ T-cell response to HIV-1 (Figure 1F). However, the cytokine pattern remained virtually unchanged for at least one year of treatment (Figures 1G, H). The subset distribution of HIV-responsive CD8^+^ T cells remained unchanged (Figure 1I), and their DI did not change significantly over the course of study. The ART-induced reversal of other differentiation and inhibitory receptors' expression was also less dramatic (Figure 1J) than that observed in total CD8^+^ T cells (Supplementary Figure 2).

### Longitudinal Analysis of CD4^+^ and CD8^+^ T-Cell Differentiation During ART

**Figure 2.**
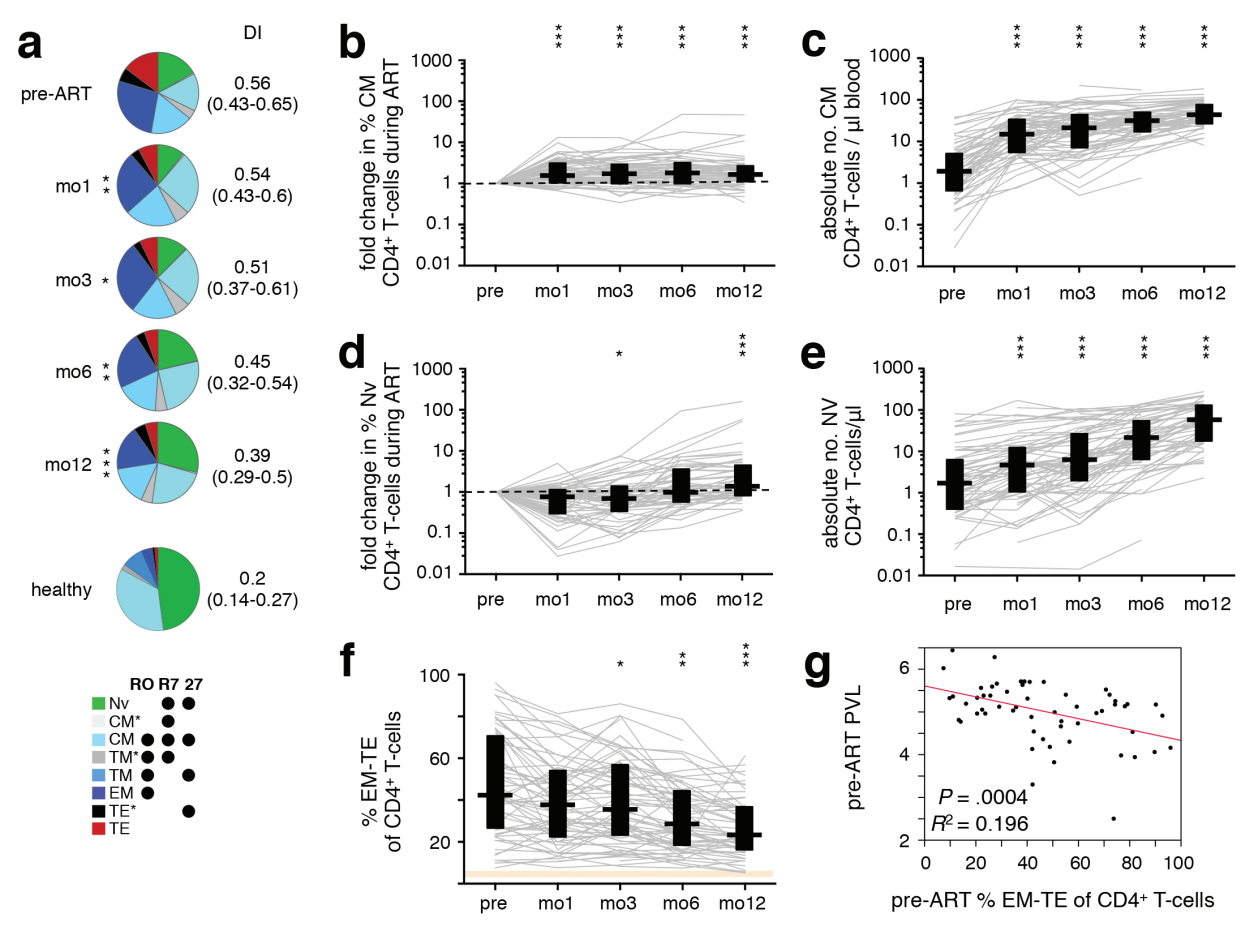
ART-induced change towards less differentiated CD4^+^ T cells. PBMC were sampled before ART, and after 1, 3, 6, and 12 months of ART. (A) The differentiation pattern was investigated in CD4^+^ T cells. Subsets were defined by expression of CD45RO (“RO”), CCR7 (“R7”) and CD27 (“27”). T_NV_–naïve; T_CM_–central memory; T_TM_–transitional memory; T_EM_–effector memory; T_TE_–terminal effector. T_CM*_, T_TM*_, and T_TE*_ are populations not classically discussed in the literature, but arise by this gating scheme; their activation phenotype and cytokine potential most closely resemble that of T_CM_, T_TM_, and T_TE_, respectively, hence their nomenclature. Differentiation indices (DI; medians and interquartile ranges) are indicated. The change in frequency over the course of treatment relative to pre-ART levels (B, D), as well as absolute cell count (C, E) of T_NV_ (B, C) and T_CM_ (D, E), and total frequency of late-differentiation (T_EM_, T_TE*_, and T_TE_) (F) CD4^+^ T-cells are shown. (G) Pre-ART PVL was plotted against pre-ART late-differentiation (T_EM_, T_TE*_, and T_TE_) CD4^+^ T cells. Graphs show development in individual patients, as well as medians and interquar-tile ranges. Corresponding interquartile ranges in healthy donors are shown where applicable (orange). All time-points were compared to corresponding pre-ART measurements: *P < 0.01, **P < 0.001, ***P < 0.0001.

We examined the evolution of T-cell differentiation over the course of ART; differentiation stage was defined by classifying cells (in rough order of maturation) as naïve (T_NV_), central memory (T_CM_ and T_CM*_), transitional memory (T_TM*_ and T_TM_), effector memory (T_EM_), or terminal effector (T_TE*_ and T_TE_) [32]. There were significant changes in the CD4^+^ T-cell subset distribution after starting ART (Figure 2A), with increasing proportions of less differentiated subsets (T_NV_, T_CM_, T_TM*_) over the course of treatment and a concomitant reduction in the proportion of highly differentiated cells (T_TE_). As previously reported, the frequency of T_CM_ was increased at mo1 (Figure 2B), prior to that of T_NV_, which became significant only at 1 year (Figure 2D). This is a reflection of the initial redistribution of memory cells [4] followed by a delayed *de novo* production and improved survival of cells [3, 5–7]. While the relative frequency of T_NV_ initially decreased due to the preferential release of memory cells from secondary lymphoid tissues, their absolute numbers increased upon introduction of ART (Figure 2E), together with that of T_CM_ (Figure 2C). The proportion of CD4^+^ T cells in late differentiation stages (T_EM_, T_TE*_, and T_TE_) steadily decreased after ART initiation (Figure 2F). Consequently, the DI of the total CD4^+^ T-cell compartment progressively decreased over the course of ART (Figure 2A). Interestingly, patients with higher pre-ART levels of late differentiation (T_EM−TE_) CD4^+^ T cells demonstrated lower pre-ART PVL (Figure 2G), but also a less dramatic recovery of CD8^+^ T-cell counts.

Nineteen of the 56 patients developed Immune Reconstitution Inflammatory Syndrome (IRIS) following ART initiation, which we showed alters CD4^+^ T-cell reconstitution kinetics, mainly by delaying T_NV_ recovery and the concomitant reduction of T_EM_, which are most evident at mo6 [18]. As a result, the inclusion of patients experiencing IRIS after commencing ART delayed the overall observed decrease of T_EM−TE_ CD4^+^ T cells (mo3 *vs.* pre-ART: *P* = .0465 IRIS, *P* = .0005 non-IRIS).

Changes in CD8^+^ T-cell subset distribution, though similar to those observed in CD4^+^ T cells, were much more subtle and only became statistically significant after many months of treatment, though some individual subsets exhibited significant changes early on (T_CM*_, T_CM_; Supplementary Figure 2A, B).

### Expression of Differentiation, Activation and Inhibitory Markers

**Figure 3.**
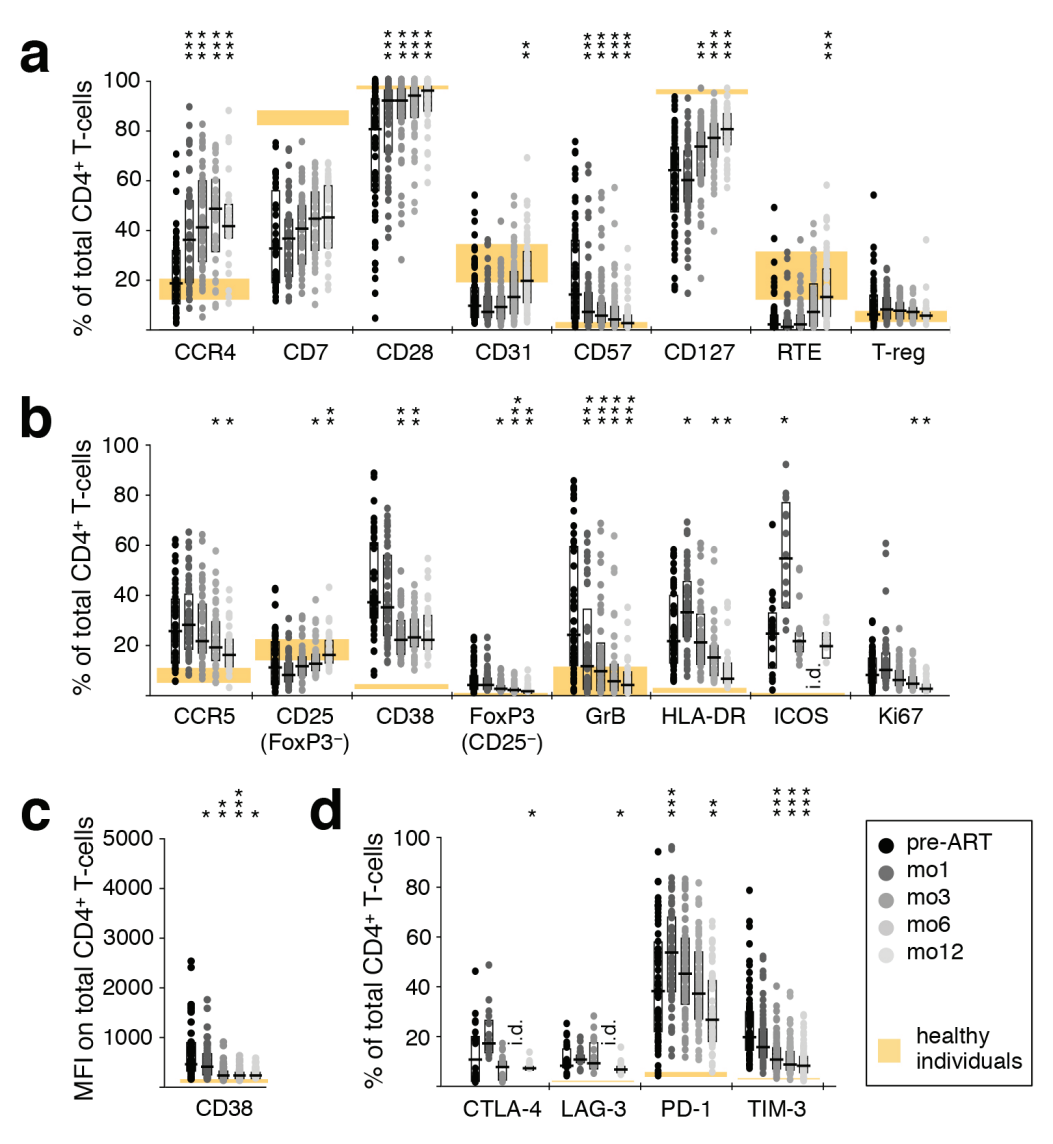
Reversal of CD4^+^ and T-cell activation during ART. Phenotypic characteristics of CD4^+^ T cells were analyzed by polychromatic flow cytometry in PBMC sampled before ART, as well as after 1, 3, 6, and 12 months of ART. (A) T-cell differentiation and subtypes; RTE–recent thymic emigrants. (B) Markers of activation; GrB–Granzyme B. (C) Mean fluorescence intensity of CD38. (D) Inhibitory receptors. Graphs show interquartile ranges, median bars, as well as individual data points. Orange areas represent the inter-quartile ranges of corresponding measurements in healthy individuals. All time-points were compared to corresponding pre-ART measurements: *P < 0.01, **P < 0.001, ***P < 0.0001. i.d.–insufficient data.

Alterations in T-cell activation phenotypes that might occur as a result of ART were comprehensively evaluated using a large range of cellular markers of T-cell differentiation, activation, and negative regulation. An ART-induced normalization of CD4^+^ T-cell differentiation was evidenced by a gradual increase in the frequency of cells expressing CD28 and CD127, paralleled by a down-regulation of the senescence marker CD57, as well as an increase in CD31^+^ cells and recent thymic emigrants (CD31^+^ CD45RO^−^ CCR7^+^) after 1 year of therapy (Figure 3(a)). A concomitant decrease in CD4^+^ T-cell activation was indicated by decreasing frequencies of CCR5^+^, CD38^+^, GrB^+^, and Ki67^+^ cells (Figure 3(b)). There was also a decrease in the MFI of CD38 expression (Figure 3(c)); elevated expression of CD38 has been closely linked to poor prognosis in HIV-1 infection [33]. The proportion of CD4^+^ T cells expressing the inhibitory receptors CTLA-4, LAG-3, or TIM-3 also declined during this time (Figure 3(d)). Compared to healthy adults, markers of CD4^+^ T-cell differentiation, activation, and expression of negative regulators normalized (or trended in that direction) over 1 year of ART.

In contrast, the frequency of cells expressing CCR4 (Figure 3A), HLA-DR, ICOS (Figure 3B), and PD-1 (Figure 3D) increased to levels significantly more disparate from those observed in healthy adults. This trend was transient for HLA-DR, ICOS, and PD-1, while for CCR4 it continued for at least one year. Note that the CCR4-expressing cells must be predominantly against specificities other than HIV, as their numbers are substantially greater than HIV-specific CD4 T cells (Figure 1).

CD8^+^ T-cell differentiation and activation normalized during treatment, but much less dramatically than that of CD4^+^ T cells, as indicated by increased CCR4, CD28, and CD127 and decreased CD57 (Supplementary Figure 2C). The inhibitory receptors CTLA-4, LAG-3, PD-1, and TIM-3, the activation markers CCR5, CD38, FoxP3, GrB, HLA-DR, ICOS, and Ki67, as well as the MFI of CD38, all declined towards normal levels (Supplementary Figure 2D-F). Interestingly, while the phenotype of CD4^+^ T cells appeared to become more similar between patients over time (reduced range of activation marker expression), this was not the case for CD8^+^ T cells.

### Co-Expression of CCR4, HLA-DR, ICOS, and PD-1

**Figure 4.**
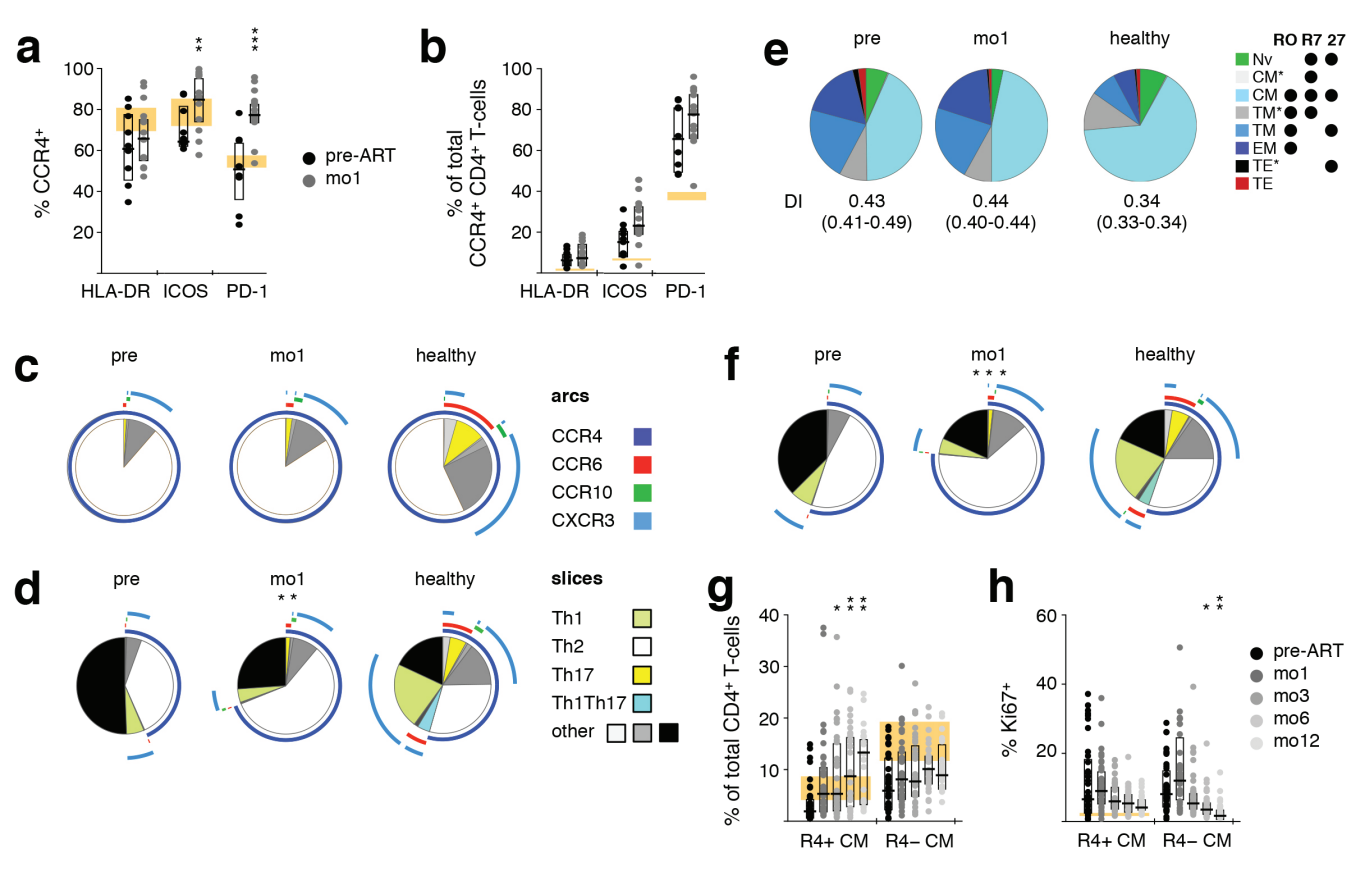
Early appearance of activated CCR4^+^ T_CM_ in peripheral blood during ART. PBMC samples taken pre-ART and after one month of ART (mo1) were analyzed to investigate co-expression of CCR4, HLA DR, ICOS, and PD-1 on CD4^+^ T cells, as well as whether these phenotypes coincide with the T_CM_ subset. (A) Frequency of CCR4^+^ cells within HLA-DR^+^, ICOS^+^, and PD-1^+^ cells. (B) Frequency of HLA-DR, ICOS, or PD-1 expressing CCR4^+^ cells. Expression of Th subset-defining chemokine receptors on CCR4^+^ (C) or non-naïve (D) CD4^+^ T cells. (E) Differentiation pattern of CCR4^+^ cells. Differentiation indices (DI; medians and interquartile ranges) are indicated below each pie. (F) Expression of Th subset-defining chemokine receptors on T_CM_ cells. (G) Proportion of CCR4^+^ T_CM_ and CCR4^−^ T_CM_ over time. (H) Expression of the proliferation marker Ki67 in CCR4^+^ T_CM_ and CCR4^−^ T_CM_ over time. Bar graphs show interquartile ranges, median bars, as well as individual data points. The interquartile range of given phenotypes (orange areas in bar charts) or average distribution patterns (pie charts) in healthy donors are shown. Mo1 measurements were compared to corresponding pre-ART values: *P < 0.01, **P < 0.001, ***P < 0.0001.

In contrast to all the other measured parameters, the expression of CCR4, HLA-DR, ICOS, and PD-1 on CD4^+^ T cells increased upon ART initiation, becoming more disparate from levels observed in healthy donors. Thus, we investigated co-expression of these molecules pre-ART and at mo1 of ART in HIV-1^+^ individuals, as well as in healthy donors. Even in healthy individuals, a large proportion of HLA-DR^+^, ICOS^+^, and PD-1^+^ CD4^+^ T cells expressed CCR4. In HIV-1^+^ patients, the CCR4^+^ fraction of ICOS^+^ and PD-1^+^ cells significantly increased shortly after commencing ART (Figure 4A). In contrast, even though the frequency of activated CCR4^+^ CD4^+^ T cells was more elevated in HIV-1^+^ patients compared to healthy donors, as measured by the expression of HLA-DR, ICOS, and PD-1, the introduction of ART did not significantly alter the proportion of activated cells (Figure 4B) or the co-expression pattern of these three activation markers (unpublished data). Taken together, these results show that the increase of HLA-DR^+^, ICOS^+^, and PD-1^+^ CD4^+^ T cells is due to the increase in activated CCR4^+^ cells.

### Th Subsets

Though the Th_1_/Th_2_ dichotomy, and wider Th-subsetting, of CD4^+^ T cells is less applicable in humans than in mice where it was first described, this system allows the identification of cellular subsets that are associated with specific functions. Hence, we here make use of the phenotypically and functionally described Th-subsets, referring to them as “Th_X_-like” where possible.

**Supplementary Figure 3.**
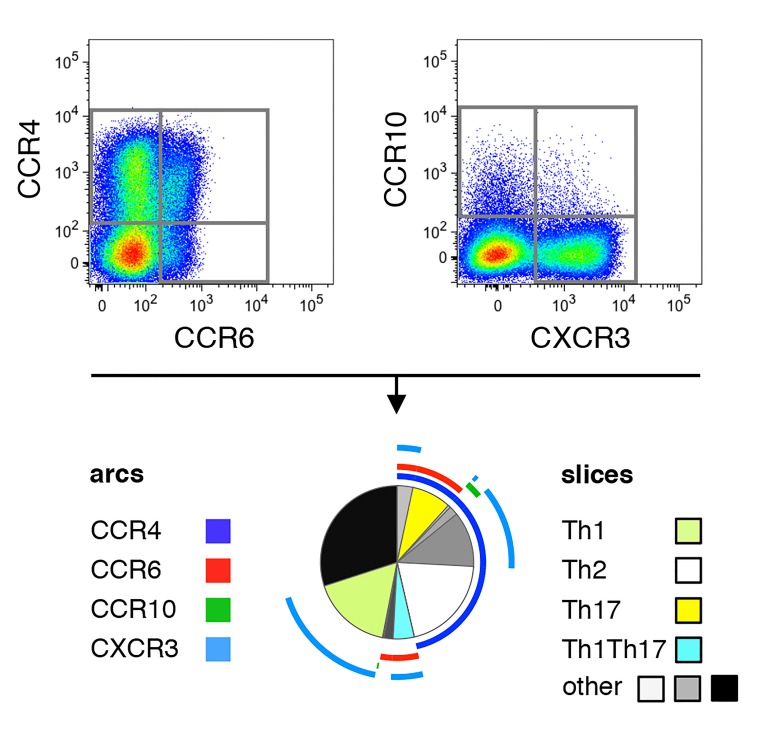
**Chemokine receptor staining and definition of Th subsets.** Gates for the expression of CCR4, CCR6, CCR10, and CXCR3 were defined within total CD4^+^ T cells. Th subsets were identified by the resulting co-expression pattern, following the gating scheme published in OMIP-017[57].

The increased prevalence of CCR4 on CD4^+^ T cells after ART initiation led us to investigate the relative representation of functionally distinct T-helper subsets prior to and during ART, as CCR4 is preferentially expressed on Th_2_-like cells. To this end, the expression pattern of CCR4, CCR6, CCR10, and CXCR3 was analyzed in order to identify cells reminiscent of Th_1_ (CCR4^−^, CCR6^−^, CXCR3^+^)[34–36], Th_2_ (CCR4^+^, CCR6^−^, CXCR3^−^) [36, 37], Th_17_ (CCR4^+^, CCR6^+^, CXCR3^−^) [38], as well as Th_1_Th_17_ cells (CCR4^−^, CCR6^+^, CXCR3^+^) capable of producing both IFN-γ and IL-17 [38] (Supplementary Figure 3). Th_9_- (CCR4^−^, CCR6^+^, CXCR3^−^) and Th_22_-like cells (CCR4^+^, CCR6^+^, CCR10^+^) [39] can also be defined using the present chemokine receptors, but these populations were too infrequent to be robustly quantifiable.

In accordance with CCR4 being preferentially expressed on Th_2_-like cells [40], the majority of CCR4^+^ CD4^+^ T cells did not express any of the other chemokine receptors analyzed. In HIV-1^+^ patients, most CCR4^+^ cells were thus defined as Th_2_-like cells, and their composition was not affected by ART (Figure 4C). However, the fraction of non-naïve CD4^+^ T cells expressing a Th_2-_like phenotype increased significantly after the induction of ART, while the proportion of Th_1_-like cells was relatively low in HIV-1^+^ patients at both time-points (Figure 4D).

### Th_2_-Like Cells and the T_CM_ Phenotype

Because a hallmark of ART-induced immune reconstitution is the early rise in the number and frequency of CD4^+^ T_CM_ [41], we investigated how the Th subsets, in particular Th_2_-like cells, correlated with this phenotype. As seen with total CD4^+^ T cells, the differentiation pattern of CCR4^+^ CD4^+^ T cells was biased towards a more differentiated population in HIV-1+ individuals. After 1 month of ART, this pattern, as well as the DI, remained unchanged, with T_CM_ representing close to 50% of CCR4^+^ cells (Figure 4E). While in healthy individuals CD4^+^ T_CM_ cells harbored balanced proportions of Th1-, Th_2_-, Th_17_-, and Th_1_Th_17_-like cells, in HIV-1^+^ patients this population was biased towards a Th_2_-like phenotype. Unlike most other changes we observed within the CD4^+^ T-cell compartment, this altered representation was dramatically exacerbated by ART initiation (Figure 4F). The co-expression pattern of HLA-DR, ICOS, and PD-1 on CD4^+^ T_CM_ closely mimicked that of CD4^+^ CCR4^+^ cells and was not affected by ART. This indicates that the observed early increases of CD4^+^ CCR4^+^ T cells and CD4^+^ T_CM_ largely identify the same population. Indeed, CCR4^+^ T_CM_ increased in frequency upon ART initiation, while CCR4−T_CM_ did not (Figure 4G). Notably, proliferation, as measured by the expression of Ki67, did not appear to be the primary mechanism for this perceived expansion (Figure 4H).

### CCR4^+^ T_CM_ are Not Genotypically Identical to Th_2_-Like Cells

**Figure 5.**
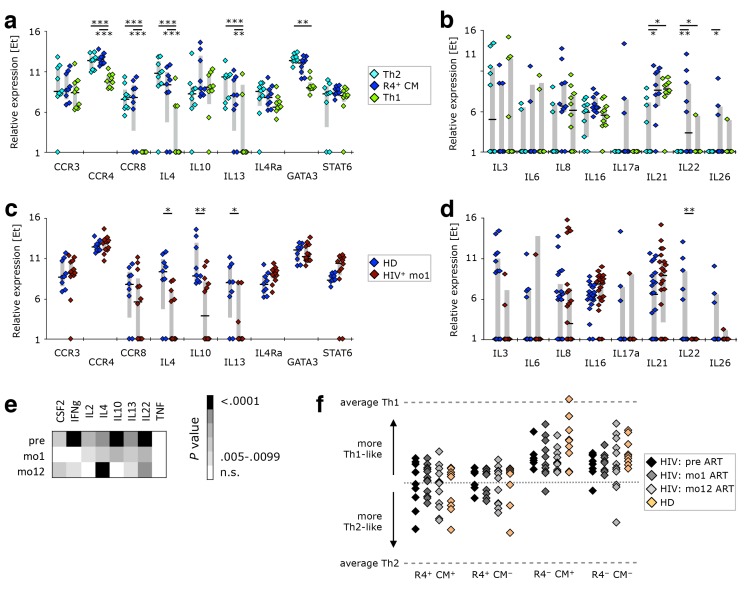
The gene expression profile of CCR4^+^ T_CM_ is different from that of Th_2_-like cells. PBMC from healthy donors, as well as cells isolated before or after 1 or 12 months of ART from HIV-1-infected adults were stained with the “sorting” panel (Supplementary Table 1). Subsets of CD4^+^ T cells were sorted as indicated in Supplementary Figure 4 and their gene expression profiles determined by multi-parametric quantitative RT-PCR. (A) Th_2_-associated and (B) other cytokine genes were compared in Th_2_-like, CCR4^+^ T_CM_, and Th_1_-like cells isolated from healthy donors. CCR4^+^ T_CM_ from HIV-1^+^ patients after 1 month of ART were compared to their counterparts from healthy donors in respect to expression of (C) Th_2_-associated or (D) other cytokine genes. (E) The expression profile of cytokine genes was investigated in nonnaïve cells. Relative expression in HIV-1-infected individuals before ART, after 1 month of ART, or 1 year of ART was compared to that in healthy donors. (F) The overall gene expression pattern of CCR4^+^ T_CM_, CCR4^+^ T_CM_^−^, CCR4^−^ T_CM_, and CCR4^−^ T_CM_^−^ cells was compared to that of Th_1_- or Th_2_-like cells sorted from healthy donors. Their calculated “Th-ness” is expressed as a point between those two extremes. Bar graphs show interquartile ranges, median bars, as well as individual data points. Statistically significant differences are indicated: *P < 0.01, **P < 0.001, ***P < 0.0001.

**Supplementary Figure 5.**
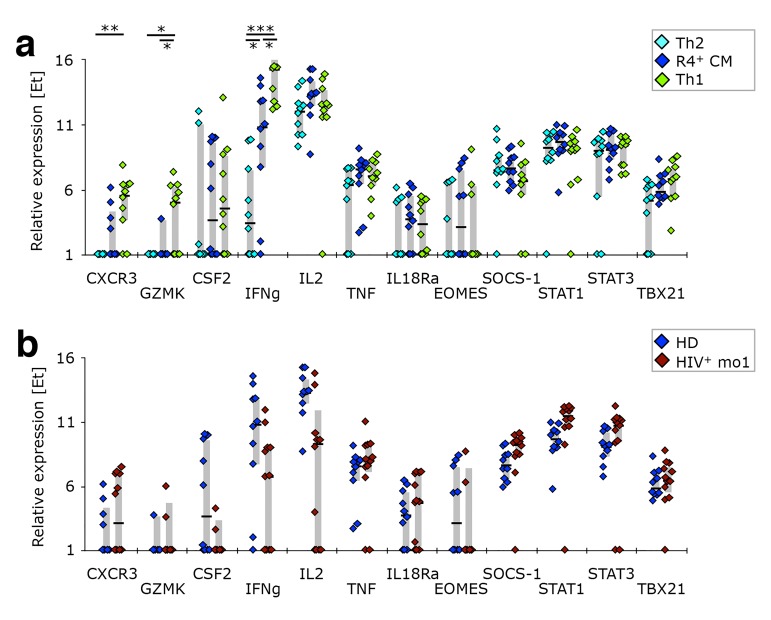
**CCR4^+^ T_CM_ resemble Th_2_-like cells in respect to their expression of Th_1_ associated genes.** PBMC from healthy donors, as well as cells isolated before or after 1 or 12 months of ART from HIV-1-infected adults were stained with the “sorting” panel (Supplementary Table 1). Subsets of CD4^+^ T cells were sorted as indicated in Supplementary Figure 4 and their gene expression profiles determined by multi-parametric quantitative RT-PCR. (A) Th_1_-associated genes were compared in Th_2-_like, CCR4^+^ T_CM_, and Th_1_-like cells isolated from healthy donors. (B) CCR4^+^ T_CM_ from HIV-1^+^ patients after 1 month of ART were compared to their counterparts from healthy donors in respect to expression of Th_1_-associated genes. Bar graphs show interquartile ranges, median bars, as well as individual data points. Statistically significant differences are indicated: * *P* < 0.01, ** *P* < 0.001, *** *P* < 0.0001.

To investigate whether CCR4^+^ T_CM_ are *bona fide* Th_2_-like cells, we compared the transcriptional profile of CCR4^+^ T_CM_, Th_2_- and Th_1_-like SEB-reactive CD4^+^ T cells from healthy donors. CCR4^+^ T_CM_ indeed closely resembled Th_2_-like cells in respect to the expression of Th_2_- (Figure 5A) and Th_1_-associated genes (Supplementary Figure 5), as well as most of the other cytokine genes investigated (Figure 5B). The only genes differentially expressed in these two cellular populations were IL21 and IL22 (both positive in CCR4^+^ T_CM_), the latter of which is typically produced by Th_17_- and Th_22_-like cells [42, 43]. CCR4^+^ T_CM_ are unlikely to be Th_22_-like, however, as their levels of IL4 and IFNg transcripts are similar to those of Th_2_- and Th_1_-like cells, respectively, neither of which is produced by Th_22_ [44]. Unlike Th_2_- or Th_1_-like cells, CCR4^+^ T_CM_ demonstrated some IL17a expression. This, together with expression of IL21 and IL22, suggests that these cells might contain an important fraction of cells with Th_17_-like functionality, even though chemokine receptor expression reveals only a small fraction of cells with a Th_17_-like phenotype (CCR4^+^ T_CM_ have a chemokine receptor expression pattern comparable to total CCR4^+^ cells (Figure 4C).

CCR4^+^ T_CM_ cells isolated from HIV-1 infected individuals one month after ART initiation had a transcriptional profile similar to that of healthy donors with respect to Th_2_- (Figure 5C) and Th_1_-associated genes (Supplementary Figure 5), as well as most other cytokine genes investigated (Figure 5D). However, there was evidence of reduced Th_2_-type cytokine transcript levels, as well as of IL22. This cytokine deficiency was not restricted to CCR4^+^ T_CM_ cells or Th_2_-associated cytokines. We found that total non-naïve cells from ART-naïve HIV-1 patients expressed lower levels of CSF2, IFNg, IL-2, IL-4, IL-10, IL-13, and IL-22 mRNA transcripts (Figure 5E).

When considering all measured genes, CCR4^+^ cell populations (both T_CM_ and non-T_CM_) were mixed in terms of having a more Th_1_- or Th_2_-like gene expression profile, while CCR4^−^ samples were mostly Th_1_-like (Figure 5F).

### Emerging CCR4^+^ T_CM_ Upon ART Induction Originate From Peripheral Tissue Sites

**Figure 6.**
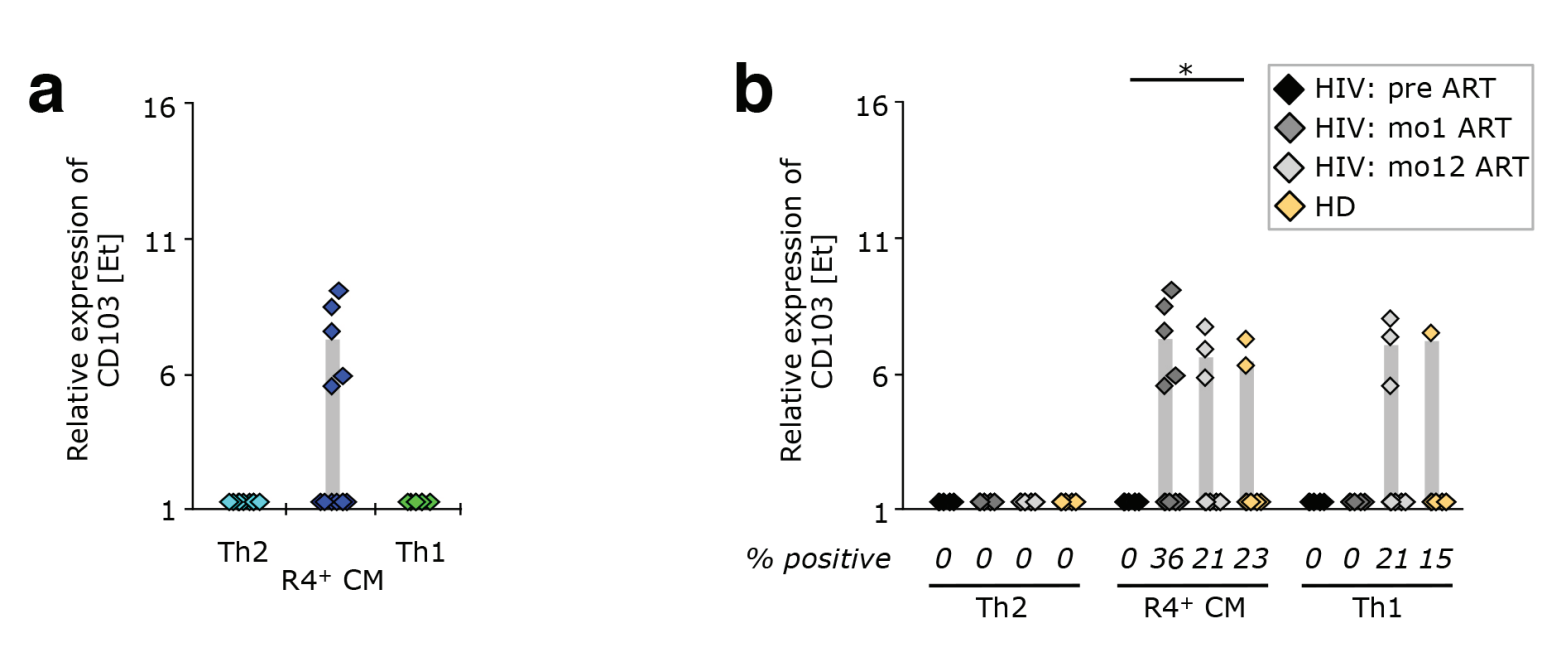
CCR4^+^ T_CM_ appear to be released from peripheral tissue sites upon ART initiation. PBMC from healthy donors, as well as cells isolated before or after 1 or 12 months of ART from HIV-1-infected adults were stained with the “sorting” panel (Supplementary Table 1). Subsets of CD4+ T cells were sorted as indicated in Supplementary Figure 4 and their gene expression profiles determined by multi-parametric quantitative RT-PCR. (A) CD103 expression on Th_2_-like, CCR4^+^ T_CM_, and Th_1_-like cells isolated after 1 month of antiretroviral therapy. (B) Expression of CD103 in CCR4^+^ T_CM_, Th_2_-, and Th_1_-like cells from healthy donor PBMC (n = 9), and longitudinal samples from HIV-1 patients (n = 12). Bar graphs show interquartile ranges, median bars, as well as individual data points. Statistically significant differences are indicated: *P < 0.01, **P < 0.001, ***P < 0.0001.

Strikingly, in PBMC from HIV-1 infected patients having received ART for one month, the inte-grin αE chain (CD103) was only found to be expressed in CCR4^+^ T_CM_ (Figure 6A). None of the other cell migration markers analyzed were found to differ between these cell populations. This is intriguing, as CD103 is part of the mucosa-homing receptor α_E_β_7_ that is widely expressed on intra-epithelial lymphocytes and lamina propria T cells, as well as on skin-resident T cells. We further investigated this phenomenon by interrogating PBMC samples obtained pre-ART, at mo1 or mo12 of ART, or from healthy donors (Figure 6B). Pre-ART, none of the T-cell subsets investigated showed any CD103 expression, whereas after 1 year of treatment expression was found in both CCR4^+^ T_CM_ and Th_1_-like cells, similar to expression detected in healthy donors. This suggests that, in healthy donors, some CCR4^+^ T_CM_ and Th_1_-like cells recirculate through peripheral tissue sites, while classical Th_2_-like cells do not.

**Supplementary Figure 6.**
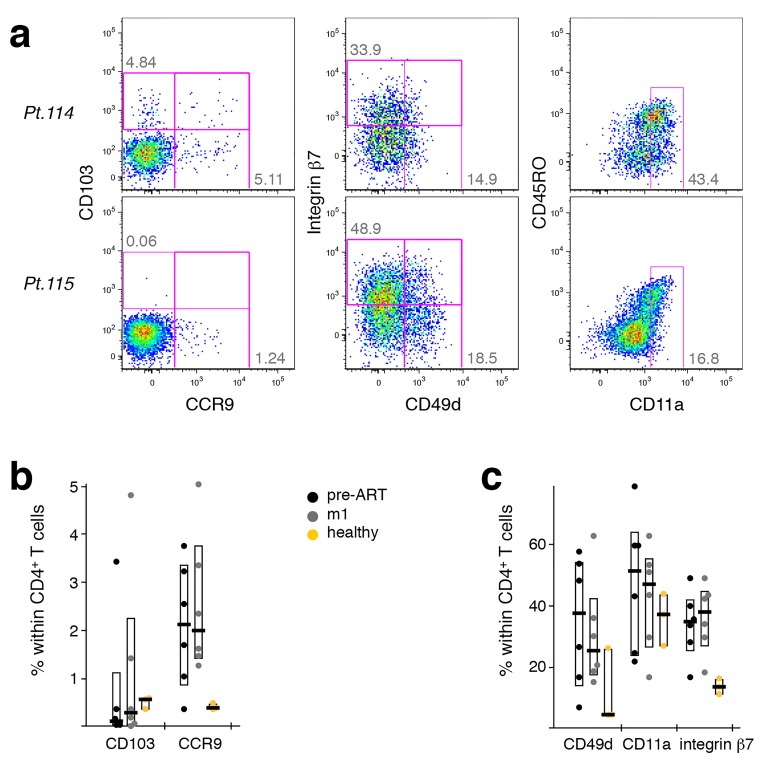
**Migration marker expression on CD4^+^ T cells.** The expression of migration markers on the cell surface of CD4^+^ T cells was investigated by flow cytometry. (A) Dot plots show the expression of CD103, CCR9, integrin β7, CD49d, and CD11a on CD4^+^ T-cells from two different patients after 1 month of ART. (B) CD103 and CCR9 expression pre-ART and after 1 month of ART compared to healthy donors. (C) CD49d, CD11a, and integrin β7 expression pre-ART and after 1 month of ART compared to healthy donors. Bar graphs show interquartile ranges, median bars, as well as individual data points. Due to the small sample size, no statistical comparison was performed.

We investigated the cell surface expression of several migration molecules on CD4^+^ T cells and found varied expression patterns between patients. HIV-1 patients appeared to have somewhat lower levels of CD103^+^ cells than healthy donors, while exhibiting higher levels of CCR9^+^ and integrin b7^+^ cells (Supplementary Figure 6). This was the case both pre-treatment and after 1 month of ART. Within CD4^+^ T cells, CD103 seemed to be preferentially expressed on CCR4^+^ cells.

There was a discordance in mRNA and protein expression of CD103, which might explain why this aberrantly expanding cellular subset was not identified previously. Down-regulation of CD103 (and other homing markers) typically occurs in a larger fraction of tissue-resident CD4^+^ T_CM_, likely heralding their release into peripheral blood (where we detected them). Maintenance of CD103 mRNA would allow for a rapid re-expression of CD103 proteins and a subsequent return to peripheral tissues.

## DISCUSSION

We characterized the phenotypic and functional T-cell dynamics in peripheral blood of severely immuno-compromised HIV-1^+^ individuals following ART. Our data confirm previous findings of an early increase in T_CM_ cells, as well as a gross reduction in overall activation levels. Almost all markers investigated, whether involved in T-cell differentiation, activation, or negative regulation, started normalizing early after ART initiation. These changes were most dramatic in CD4^+^ T cells, but mirrored by similar changes in CD8^+^ T cells, which retained a larger range of individual marker expression than CD4^+^ T cells.

In contrast to the general trend towards normalization, CD4^+^ T-cell activation (HLA-DR, ICOS, PD-1) initially increased in peripheral blood before gradually decreasing, too. CCR4^+^ cells demonstrated a more sustained increase, reflecting a skewing towards a Th_2_-like environment early after ART initiation, and a further deregulation away from the phenotype of healthy donors.

A shift from cells with Th_1_- to those with Th_2_-like functionality has previously been suggested to occur during HIV-1 infection [45–47]. It has been reported that long-term non-progressors exhibit a Th_1_-like cytokine profile, while progressors exhibit a Th_2_-like cytokine profile [47], and that increasing viral loads correlate with lower cytoplasmic levels of IL-2 and IFN-γ and concomitant increases in IL-4 and IL-10 levels after stimulation with PMA/ionomycin [46]. Also, certain IL-4Rα SNPs linked to IL-4 hypo-responsiveness may be associated with slower HIV-1 disease progression [48]. The Th_1_- to Th_2_-like switch observed during disease progression was suggested to be at least partially due to an initial selective loss of CCR5^+^ Th_1_-like cells [49]. Our data confirm the presence of an overwhelming predominance of phenotypically Th_2_-like cells during very advanced (< 200 CD4^+^ T cells/μl) HIV-1 infection.

Early studies have shown that CD4^+^ T_CM_ are released from tissues into the bloodstream early after commencing ART [4], leading to the initial boost in CD4 counts. The present data suggest that these CD4^+^ T_CM_ are primarily CCR4^+^. Thus, the CCR4^+^ T_CM_ cells appearing in the PBMC upon ART do not reflect a phenotypic change of cells preexisting in the blood stream—that is, an ART-induced change in the Th environment—but rather the appearance of a cell type previously sequestered in the tissues [50, 51].

This is supported by our findings that, after one month of therapy, CD103 transcripts were specifically expressed by CCR4^+^ T_CM_, while no expression was detected in any of the subsets investigated prior to ART. The α_E_ integrin chain (CD103) is typically found on intra-epithelial lymphocytes, allowing them to home to and circulate through mucosal sites [52]. It represents a rare transcript in peripheral blood CD4^+^ T cells, and indicates that ART induces the release of CCR4^+^ T_CM_ from tissues.

Early after ART commencement, while PVL levels were still in the decline, these CCR4^+^ T_CM_ were highly activated, expressing ICOS, HLA-DR, and PD-1. As the PVL were suppressed to undetectable levels at mo3 of ART, the activation of CCR4^+^ T_CM_ also leveled off. However, there was no normalization of the Th_2_-like phenotype (% CCR4^+^ CD4^+^ T cells), even 1 year after ART.

Gene expression analyses confirmed that the CCR4^+^ T_CM_ cells are largely comparable to classic Th_2_ cells in their Th_1_- and Th_2_-associated transcriptome. However, the fact that CD103 mRNA was detected in CCR4^+^ T_CM_ but not Th_2_-like cells indicated that the cells released from tissues are not classical Th_2_-like cells. Expression of IL21 and IL22 (and to a lesser degree IL17a) transcripts, cytokines not typically associated with Th_2_-like cells, suggests that the CCR4^+^ T_CM_ contains a fraction of cells with a Th_17_-like functionality. Such cells are important in controlling bacterial infections at mucosal surfaces such as the gut and lungs [53]; the measured Th_17_-like functionality correlates well with expression of CD103 mRNA, the protein product of which has been implicated with homing to gut and skin [54].

The recovery kinetics of naïve and memory CD4^+^ T cells on ART have been shown to differ depending on the extent of a patient's CD4^+^ T-cell loss at the time of ART initiation [9]. Therefore, the present findings might not apply to all HIV-1^+^ individuals on ART, as we focused our study on severely immuno-compromised patients (< 200 CD4^+^ T cells/μl). In fact, we found an inverse correlation between CD4^+^ (*P* = 0.0008) or CD8^+^ (*P* = 0.0037) T-cell recovery and the CD4^+^ T-cell count pre-ART, reflecting a greater change in T-cell numbers experienced by those patients starting with a more severe T-cell depletion. Furthermore, individuals with lower pre-ART PVL were found to start with more differentiated CD4^+^ T cells and to exhibit a more rapid drop in late differentiation cells.

However, even though a substantial immune reconstitution occurs in the peripheral blood, it has been demonstrated that the mucosal immune system is more recalcitrant [55, 56], with CD4^+^ T cells expressing CCR5 and/or CXCR4 remaining preferentially depleted [56].

Overall we found that even in these very advanced HIV-1^+^ patients the CD4^+^ and CD8^+^ T-cell compartments in peripheral blood slowly revert towards a more “healthy” phenotype, with an overall reduction in the expression of activation markers and molecules associated with inhibition of cellular functions, as well as an upregulation of factors associated with T-cell homeostasis and a more balanced immune system. For the most part, these changes commence early after ART initiation. An exception was an ART-induced increase in the frequency of initially highly activated peripheral CCR4^+^ T_CM_ cells. This reflects a redistribution of these cells from peripheral tissue sites to peripheral blood, caused by a reduction in the viral burden, as highlighted by their expression of mRNA transcripts of the gut- and skin-homing integrin CD103. However, even 1 year post-therapy, the immune system maintains an unusually Th_2_-biased composition, potentially underlying continued immunodeficiency in the presence of higher CD4^+^ T-cell counts.

## Figures and Tables

**Supplementary Figure 4.**
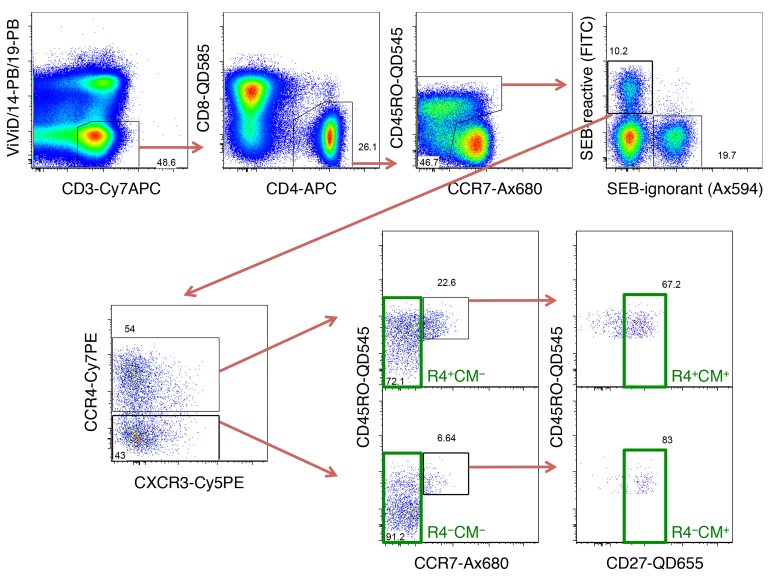
**T-cell subsets sorted for gene expression analysis.** PBMC were stimulated with SEB for 3 hours, before sorting seven CD4^+^ T-cell subsets from SEB-reactive cells as indicated in this gating scheme. After identifying live CD3^+^ cells within singlet, aggregate-negative lymphocytes, non-naive CD4^+^ T cells were selected by excluding CD45RO^−^ CCR7^+^ cells. Within these, T cells expressing TCRVβ12, -Vβ14 or -Vβ17 are known to react to SEB, so FITC-conjugated Abs were used for these three TCR-Vβ chains. Within such SEB-reactive cells, CCR4^+^ and CCR4− cells were selected and individually gated for T_CM_ (CD45RO^+^ CCR7^+^ CD27^+^) or T_CM_^−^ subsets. Further, total SEB-reactive CD4^+^ T cells, as well as stringently gated Th_1_- (CCR4^−^, CCR6^−^, CCR10^−^, CXCR3^+^) and Th_2_-like cells (CCR4^+^ CCR6^−^ CCR10^−^ CXCR3^−^) were also sorted.

**Supplementary Table 1.** Reagent panels used for flow cytometric analysis

Detector*	Fluorochroe	Reagents used in panel						
		Functional	Activation	Inhibitory	T-reg	Th subset	Sorting	Migration
*V450*	ViViD	Dead cells	Dead cells	Dead cells	Dead cells	Dead cells	Dead cells	Dead cells
	PacBlu	CD14 / CD19	CD14 / CD19	CD14 / CD19	CD14 / CD19	CD14 / CD19	CD14 / CD19	CD14 / CD19
*V545*	QD545	CD45RO	CD45RO	CD45RO	CD45RO	CD45RO	CD45RO	
*V565*	Biotin + SA-QD565 / QD565	CD57						CD49
*V585*	QD585	CD8	CD8	CD8	CD8	CD8	CD8	CD8
*V605*	Biotin + SA-QD605 / BV605	PD-1	PD-1	PD-1	CCR5	PD-1	CCR6	CCR6
*V655*	QD655	CD27		CD27	CD27	CD27	CD27	CD27
*V705*	QD705	CD7	CD57	CD57	CD57			CD11a
*V800*	QD800	CD4	CD4	CD4	CD4	CD4		CD4

*B515*	Ax488 / FITC	IFN-γ	Ki67	Ki67	Ki67	CCR6	TCR-Vβ^12, 14, 17^	CD103

*G560*	PE	TIM-3	CD38	LAG-3	CD127	CCR10	CCR10	CCR10
*G610*	Ax594	TNF	CCR7		CD45RA		TCR-Vβ^1, 2, 7, 13.6, 16, 22^	CD3
*G660*	PE-Cy5	CD28	HLA-DR		FoxP3	CXCR3	CXCR3	CXCR3
*G710*	PE-Cy5.5	CD127				HLA-DR		Integrin β7
*G780*	PE-Cy7	CD31	CCR5	ICOS	CD25	ICOS	CCR4	CCR4

*R660*	APC	IL-2	Granzyme B	CTLA-4	CCR4	CCR4	CD4	CCR9

*R710*	Ax680 / Ax700	CCR7	CD27	CCR7	CCR7	CCR7	CCR7	CCR7
*R780*	APC-Cy7	CD3	CD3	CD3	CD3	CD3	CD3	

ViViD indicates LIVE/DEAD fixable violet dead cell stain; PacBlu, pacific blue; QD, quantum dot; SA, streptavidin; BV, brilliant violet; Ax, alexa; FITC, fluorescein isothiocyanate; PE, R-phycoeryhtrin; Cy, cyanine; APC, allophycocyanin.

* Detectors are coded by laser colour (V indicates violet; B, blue; G, green; R, red) and mean wavelength measured.

**Supplementary Table 2.** Genes investigated by quantitative RT-PCR

	Gene Symbol	Other names	Gene Name	Assay Catalogue #
*Cytokines*	CSF2	GM-CSF	(granulocyte-macrophage) colony stimulating factor 2	Hs00929873_m1
	IFNg		interferon gamma	Hs00174143_m1
	IL2		interleukin 2	Hs00174114_m1
	IL3		interleukin 3 (colony-stimulating factor for multiple cell types)	Hs99999081_m1
	IL4		interleukin 4	Hs00929862_m1
	IL5		interleukin 5 (colony-stimulating factor for eosinophils)	Hs99999031_m1
	IL6		interleukin 6 (interferon, beta 2)	Hs00985639_m1
	IL8		interleukin 8	Hs99999034_m1
	IL9	HP40, P40	interleukin 9	Hs00914237_m1
	IL10		interleukin 10	Hs00961622_m1
	IL13		interleukin 13	Hs99999038_m1
	IL16		interleukin 16 (lymphocyte chemo-attractant factor)	Hs00189606_m1
	IL17a		interleukin 17A	Hs00174383_m1
	IL21		interleukin 21	Hs00222327_m1
	IL22		interleukin 22	Hs00220924_m1
	IL26		interleukin 26	Hs00218189_m1
	LTA	TNFb	lymphotoxin alpha (TNF superfamily, member 1)	Hs00236874_m1
	LTB	TNFc	lymphotoxin beta (TNF superfamily, member 3)	Hs00242739_m1
	TGFB1		transforming growth factor, beta 1	Hs00998133_m1
	TGFB2		transforming growth factor, beta 2	Hs01548875_m1
	TNF	TNFa	tumor necrosis factor	Hs00174128 m1
*Cytokine receptors*	IL2Ra	CD25	interleukin 2 receptor, alpha	Hs00907778_m1
	IL2Rb	CD122	interleukin 2 receptor, beta	Hs01081697_m1
	IL3RA	CD123	interleukin 3 receptor, alpha (low affinity)	Hs00608141_m1
	IL4Ra	CD124	interleukin 4 receptor	Hs00166237_m1
	IL5Ra	CD125	interleukin 5 receptor, alpha	Hs01064360_m1
	IL6R	CD126	interleukin 6 receptor	Hs01075667_m1
	IL6ST	gp130	interleukin 6 signal transducer (oncostatin M receptor)	Hs00174360_m1
	IL7R	CD127	interleukin 7 receptor	Hs00233682_m1
	IL10Ra	CD210	interleukin 10 receptor, alpha	Hs00155485_m1
	IL12RbI	CD212	interleukin 12 receptor, beta 1	Hs00538167_m1
	IL12RbII		interleukin 12 receptor, beta 2	Hs01548202_m1
	IL18Ra	IL-18R1	interleukin 18 receptor 1	Hs00977691_m1
	IL21R		interleukin 21 receptor	Hs00222310_m1
	TGFBR1		transforming growth factor, beta receptor 1	Hs00610318_m1
	TGFBR3	BGCAN	transforming growth factor, beta receptor 3	Hs01114253 m1
*Migration-associated receptors*	CCR1	CD191	C-C motif chemokine receptor 1	Hs00174298_m1
	CCR2	CD192	C-C motif chemokine receptor 2	Hs00356601_m1
	CCR3	CD193	C-C motif chemokine receptor 3	Hs00266213_s1
	CCR4	CD194	C-C motif chemokine receptor 4	Hs99999919_m1
	CCR5	CD195	C-C motif chemokine receptor 5	Hs00152917_m1
	CCR6	CD196	C-C motif chemokine receptor 6	Hs00171121_m1
	CCR7	CD197	C-C motif chemokine receptor 7	Hs99999080_m1
	CCR8		C-C motif chemokine receptor 8	Hs00174764_m1
	CCR10		C-C motif chemokine receptor 10	Hs00706455_s1
	CD103	ITGAE	integrin, alpha E (human mucosal lymphocyte antigen 1; alpha polypeptide)	Hs00559580_m1
	CXCR1	IL-8R1	C-X-C motif chemokine receptor 1	Hs00174146_m1
	CXCR2	IL-8R2	C-X-C motif chemokine receptor 2	Hs01011557_m1
	CXCR3	CD183b, MIGR	C-X-C motif chemokine receptor 3	Hs00171041_m1
	CXCR4		C-X-C motif chemokine receptor 4	Hs00976734_m1
	CXCR5		C-X-C motif chemokine receptor 5	Hs00173527_m1
	CXCR6		C-X-C motif chemokine receptor 6	Hs00174843_m1
	GPR44	CRTH2, CD294	G protein-coupled receptor 44	Hs00173717 m1
*Prolif.*	MKI67	Ki67	antigen identified by monoclonal antibody Ki67	Hs01032443_m1
	PCNA		proliferating cell nuclear antigen	Hs00696862_m1
*Chemokines*	CCL2	MCP1	C-C motif chemokine ligand 2	Hs00234140_m1
	CCL3	MIP-1α	C-C motif chemokine ligand 3	Hs00234142_m1
	CCL4	MIP-1β	C-C motif chemokine ligand 4	Hs01031494_m1
	CCL5	RANTES	C-C motif chemokine ligand 5	Hs00174575_m1
	CXCL9	MIG	C-X-C motif chemokine ligand 9	Hs00171065_m1
	CXCL10		C-X-C motif chemokine ligand 10	Hs00171042_m1
	CXCL11	I-TAC	C-X-C motif chemokine ligand 11	Hs00171138_m1
	CXCL13	BLC, BCA-1	C-X-C motif chemokine ligand 13	Hs00757930 m1
*Cytolysis*	GNLY		granulysin	Hs00246266_m1
	GZMA	CTLA3	granzyme A (granzyme 1; cytotoxic T-lymphocyte associated serine esterase 3)	Hs00989184_m1
	GZMB	CTLA1	granzyme B (granzyme 2; cytotoxic T-lymphocyte associated serine esterase 1)	Rh02621701_m1
	GZMH	CTSG	granzyme H (cathepsin Glike 2; protein hCCPX)	Hs00277212_m1
	GZMK	TRYP2	granzyme K (granzyme 3; tryptase II)	Hs00157878_m1
	GZMM	MET1	granzyme M (lymphocyte metase 1)	Hs00193417_m1
	PRF1	Perforin	perforin 1 (pore forming protein)	Hs00169473 m1
*Transcription factors*	ARNT		aryl hydrocarbon receptor nuclear translocator	Hs00231048_m1
	EOMES	TBR2	eomesodermin	Hs00172872_m1
	FOXP3		forkhead box P3	Hs00203958_m1
	GATA3		GATA binding protein 3	Hs00231122_m1
	RORC	RORgT	RAR-related orphan receptor C	Hs01076112_m1
	SOCS-1		suppressor of cytokine signaling 1	Hs00705164_s1
	STAT1		signal transducer and activator of transcription 1	Rh02899274_m1
	STAT3		signal transducer and activator of transcription 3 (acute-phase response factor)	Hs01047580_m1
	STAT4		signal transducer and activator of transcription 4	Rh02896026_m1
	STAT5A		signal transducer and activator of transcription 5A	Rh02844611_m1
	STAT6		signal transducer and activator of transcription 6	Hs00598625_m1
	TBX21	T-bet	Tbox 21	Hs00894392 m1
*Activation*	CD38		CD38 molecule	Hs01120071_m1
	CTLA4		cytotoxic T-lymphocyte associated protein 4	Hs03044418_m1
	HLADRA		major histocompatibility complex, class II, DR alpha	Hs00219575_m1
	HAVCR1	TIM-1	hepatitis A virus cellular receptor 1	Rh02863844_m1
	HAVCR2	TIM-3	hepatitis A virus cellular receptor 2	Hs00958623_m1
	LAG3	CD223	lymphocyte activation gene 3	Hs00158563_m1
	PD1	PDCD1	programmed cell death 1	Hs01550088 m1
*Costimulation*	ICOS		inducible T-cell costimulator	Hs00359999_m1
	TNFRSF1	RANK	tumor necrosis factor receptor superfamily, member 11a, NFKB activator	Hs00187192_m1
	TNFRSF4	Ox40	tumor necrosis factor receptor superfamily, member 4	Hs00533968_m1
	TNFRSF9	CD137, 4-1BB	tumor necrosis factor receptor superfamily, member 9	Hs00155512_m1
	TNFSF10	TRAIL	tumor necrosis factor (ligand) superfamily, member 10	Hs00921974_m1
	TNFSF13B	BAFF	tumor necrosis factor (ligand) superfamily, member 13b	Hs00198106_m1
	TNFSF14	LIGHT	tumor necrosis factor (ligand) superfamily, member 14	Hs00187011 m1
